# Emergency diagnosis of cancer and previous general practice consultations: insights from linked patient survey data

**DOI:** 10.3399/bjgp17X690869

**Published:** 2017-04-25

**Authors:** Gary A Abel, Silvia C Mendonca, Sean McPhail, Yin Zhou, Lucy Elliss-Brookes, Georgios Lyratzopoulos

**Affiliations:** University of Exeter Medical School (Primary Care), Exeter and Cambridge Centre for Health Services Research, Institute of Public Health, University of Cambridge, Cambridge.; Cambridge Centre for Health Services Research, Institute of Public Health, University of Cambridge, Cambridge.; Public Health England National Cancer Registration and Analysis Service, London.; Cambridge Centre for Health Services Research, Institute of Public Health, University of Cambridge, Cambridge.; Public Health England National Cancer Registration and Analysis Service, London.; Department of Behavioural Science and Health, University College London, London and Cambridge Centre for Health Services Research, Institute of Public Health, University of Cambridge, Cambridge.

**Keywords:** cancer, diagnosis, emergency, opportunities, primary care

## Abstract

**Background:**

Emergency diagnosis of cancer is common and aetiologically complex. The proportion of emergency presenters who have consulted previously with relevant symptoms is uncertain.

**Aim:**

To examine how many patients with cancer, who were diagnosed as emergencies, have had previous primary care consultations with relevant symptoms; and among those, to examine how many had multiple consultations.

**Design and setting:**

Secondary analysis of patient survey data from the 2010 English Cancer Patient Experience Survey (CPES), previously linked to population-based data on diagnostic route.

**Method:**

For emergency presenters with 18 different cancers, associations were examined for two outcomes (prior GP consultation status; and ‘three or more consultations’ among prior consultees) using logistic regression.

**Results:**

Among 4647 emergency presenters, 1349 (29%) reported no prior consultations, being more common in males (32% versus 25% in females, *P*<0.001), older (44% in ≥85 versus 30% in 65–74-year-olds, *P*<0.001), and the most deprived (35% versus 25% least deprived, *P* = 0.001) patients; and highest/lowest for patients with brain cancer (46%) and mesothelioma (13%), respectively (*P*<0.001 for overall variation by cancer site). Among 3298 emergency presenters with prior consultations, 1356 (41%) had three or more consultations, which were more likely in females (*P*<0.001), younger (*P*<0.001), and non-white patients (*P* = 0.017) and those with multiple myeloma, and least likely for patients with leukaemia (*P*<0.001).

**Conclusion:**

Contrary to suggestions that emergency presentations represent missed diagnoses, about one-third of emergency presenters (particularly those in older and more deprived groups) have no prior GP consultations. Furthermore, only about one-third report multiple (three or more) consultations, which are more likely in ‘harder-to-suspect’ groups.

## INTRODUCTION

Evidence from several countries documents that many patients with cancer are diagnosed through an emergency presentation (hereafter, the term ‘emergency presenters’ is used for these patients).^1^^–^4 As emergency presentations are associated with poorer survival and worse patient experience, reducing their frequency is desirable, but how to achieve such reductions is uncertain.^5^^–^8 Complex aetiologies, reflecting different disease (tumour), patient, and healthcare factors, often in combination, are likely to be implicated.^9^^–^11 Prior evidence indicates that older patients and patients who are more socioeconomically deprived are at substantially higher risk of diagnosis of cancer as an emergency.^1^^,^^3^^,^^4^ An ecological study indicates a degree of variation by certain general practice characteristics, but overall there is very limited evidence about whether and how diagnoses of cancer through an emergency presentation can be avoided.^11^^,^^12^

A key consideration is whether emergency presentations were preceded by patient contact with the healthcare system. Some emergency presenters would have had no such prior contact. This can occur either when dramatic (life-threatening) clinical presentations are preceded by minimal or short-lived symptoms, making emergency presentations practically unavoidable (indeed representing optimal care); or when patients have experienced, often mild, symptoms for a long time but have not sought medical help until sudden changes in the nature or severity of their symptoms prompted them to do so in an emergency context.^9^^,^^10^ Practical (for example, access), cognitive (for example, symptom awareness), or emotional (for example, fear of cancer diagnosis) factors may all act as barriers to help seeking.

Other emergency presenters would have sought medical help for their symptoms previously (often in primary care), but either referrals or investigations were not initiated; or a diagnostic process was instigated but sudden subsequent changes in symptom nature or severity led to an emergency presentation.^9^^,^^10^ Additionally, emergency presentations may result from contact with a GP who then directly refers the patient as an emergency to the hospital.^13^

As also highlighted by the findings of a recent in-depth review of the diagnosis of cancer as an emergency, evidence on the proportion of emergency presenters with and without previous consultations is very limited.^11^ This study aimed to examine variation (by patient characteristic and cancer site) in the presence and number of prior consultations with a GP among emergency presenters, to provide insights into aetiological mechanisms responsible for such presentations.

**How this fits in**

Diagnosis of cancer as an emergency has been considered to represent ‘a failure of primary care’. Evidence to support such assertions is, however, limited. A notable minority of all emergency presenters have no prior contact with primary care, particularly males, older, and more deprived patients, and those with brain cancer. Among emergency presenters who have seen a GP, multiple consultations occur in a minority and are driven by diagnostic difficulty.

## METHOD

### Data

A secondary analysis was performed of data from the 2010 English Cancer Patient Experience Survey (CPES), previously linked to information about diagnostic ‘route’ (see below) as part of a national report on cancer patient experience.^8^^,^^14^

The patient survey was commissioned by the UK Department of Health and carried out by Quality Health, a specialist survey provider.^12^ The sampling frame included patients aged ≥16 years with a cancer diagnosis, seen as inpatients or day cases in English NHS hospitals during January–March 2010. After vital status checks, patients were sent the survey questionnaire by post a few weeks after their treatment, with up to two reminders for non-responders.

Analyses were restricted to responders who were emergency presenters, based on data linkage with the Routes to Diagnosis dataset, carried out previously by the Public Health England (former) National Cancer Intelligence Network to support public reporting of data on cancer patient experience (Appendix 1).^8^ Diagnostic ‘routes’ represent different care pathways to cancer diagnosis.^1^ The emergency presentation route denotes new diagnosis of cancer following accident and emergency department attendance, emergency hospital admission, emergency between-hospital transfer, or emergency GP referral (operational definitions have been detailed previously).^1^

#### Outcomes

Variation was examined in respect of two outcomes: First, prior consultation status, that is whether emergency presenters had no prior GP consultations; and, second, ‘multiple’ consultation status, that is whether emergency presenters who had seen a GP had three or more consultations. Both outcomes were defined using information from the first survey question: *‘Before you were told you needed to go to hospital about cancer, how many times did you see your GP (family doctor) about the health problem caused by cancer?’*. Informative response options included ‘*None — I did not see my GP before going to hospital’* (denoting *‘no prior GP consultation’* in the analysis) and *‘Once’, ‘Twice’, ‘Three or four times’,* and *‘Five or more times’* (the latter two categories used to denote ‘three or more’ consultations among patients reporting at least one consultation).

The latter outcome was dichotomised (three or more versus one to two consultations), consistent with public reporting conventions for this survey, and because some repeat (second) appointments are generated by the need to review the results of investigations ordered at an initial consultation. The number of pre-diagnostic consultations is associated with the primary care interval (number of days between presentation in primary care and referral), with primary care intervals of 34, 47, and 97 days for the average patient with three, four, and ‘five or more’ consultations, respectively.^15^

#### Exposures

These included patients’ age group, sex, deprivation group, and cancer site using the International Classification of Diseases 10th edition (based on hospital records information included in the CPES dataset); and self-reported ethnic group (derived from survey responses) using a binary white/non-white variable because of the small numbers in minority ethnic groups. Deprivation groups were defined using quintiles of the English Index of Multiple Deprivation 2007 score based on patients’ postcodes.

#### Sample derivation

Of an initial total of 67 713 responders, diagnostic route information was available for 56 363 (83%). For practical reasons, analyses were restricted to emergency presenters with one of the 18 most common cancers. The final analysis sample included 4647 emergency presenters with complete information for all covariates (Appendix 2).

#### Analysis

Prior hypotheses regarding variation in both outcomes are shown in Box 1. Logistic regression models were used to estimate crude and adjusted odds ratios (ORs). The adjusted models included cancer site and sociodemographic variables.

Box 1. **Prior hypotheses**

It was a *priori* hypothesised that the symptom signature of different cancers would be the main driver of variation by cancer. Specifically, it was hypothesised that in respect of symptom signatures:

- Emergency presenters with cancers that often present acutely with symptoms deemed a clinical emergency (for example, seizures in patients with brain cancer) would probably have low proportions of prior consultations.
- Emergency presenters with ‘harder-to-suspect’ cancers (for example, multiple myeloma) would probably have consulted multiple times.

Additionally, it was hypothesised that emergency presenters with leukaemia (where emergency presentations may result from abnormal findings in full blood count tests carried out in primary care), who were prior consultees, would probably not have consulted multiple times.

The survey sample is drawn from patients with cancer with recent hospital treatment rather than incident cases. Variations in treatment modality by cancer site distort the sample’s composition compared with incident cases.^16^ Further sample distortions occur because of post-sampling mortality and survey non-response.^5^ Therefore, there are likely compositional differences between the analysis sample and the population of prior interest (incident emergency presenters). To account for this, when estimating overall percentages post-stratification weights were produced, derived using the complete (as opposed to linked) ‘Routes-to-diagnosis’ 2006–2010 dataset from a previous study (Appendix 3).^4^

As all variables used in the derivation of weights were included in the adjusted models, weighting was not used in the regression analysis. Assuming the models are correctly specified, estimated OR should be unbiased and standard errors would be smaller than if weights had been included. As a sensitivity analysis, the regression analysis was repeated including weights.

## RESULTS

The characteristics of the 4647 emergency presenters included in the analysis are shown in Table 1. Older and more deprived emergency presenters were under-represented in the analysis sample, with only small differences by sex. Patients with haematological cancers (multiple myeloma, Hodgkin and non-Hodgkin lymphomas, and leukaemia) were over-represented and those with lung and pancreatic cancer under-represented (Appendix 3).

**Table 1.** Predictors of no prior GP consultation among emergency presenters (*n* = 4647)

	**Emergency presenters, *N***	**Emergency presenters without prior consultations, *n***	**% (*n*/*N*)**	**Unadjusted OR (95% CI)**	**Adjusted OR (95% CI)^a^**	***P*-value^b^**
**Cancer site**						
Brain	173	80	46.2	1.84 (1.31 to 2.57)	2.08 (1.47 to 2.94)	<0.001
Renal	102	45	44.1	1.68 (1.11 to 2.56)	1.71 (1.12 to 2.62)	
Endometrial	57	22	38.6	1.34 (0.77 to 2.34)	1.54 (0.88 to 2.72)	
Breast	156	54	34.6	1.13 (0.79 to 1.62)	1.42 (0.97 to 2.07)	
Rectal	235	93	39.6	1.40 (1.03 to 1.89)	1.37 (1.01 to 1.86)	
Oesophageal	86	33	38.4	1.33 (0.84 to 2.11)	1.16 (0.73 to 1.85)	
Stomach	116	43	37.1	1.26 (0.84 to 1.89)	1.11 (0.74 to 1.67)	
Lung	389	134	34.4	1.12 (0.86 to 1.45)	1.11 (0.85 to 1.44)	
Colon	752	240	31.9	Reference	Reference	
Bladder	264	92	34.8	1.14 (0.85 to 1.53)	0.97 (0.72 to 1.31)	
Prostate	209	73	34.9	1.15 (0.83 to 1.58)	0.93 (0.67 to 1.30)	
Leukaemia	519	130	25.0	0.71 (0.55 to 0.92)	0.81 (0.62 to 1.05)	
Multiple myeloma	513	119	23.2	0.64 (0.50 to 0.83)	0.66 (0.51 to 0.86)	
Non-Hodgkin lymphoma	514	112	21.8	0.59 (0.46 to 0.77)	0.63 (0.48 to 0.81)	
Pancreatic	118	20	16.9	0.44 (0.26 to 0.72)	0.45 (0.27 to 0.74)	
Ovarian	324	44	13.6	0.34 (0.24 to 0.48)	0.41 (0.29 to 0.60)	
Hodgkin lymphoma	59	7	11.9	0.29 (0.13 to 0.64)	0.35 (0.15 to 0.80)	
Mesothelioma	61	8	13.1	0.32 (0.15 to 0.69)	0.30 (0.14 to 0.64)	

**Sex**						
Male	2526	819	32.4	Reference	Reference	<0.001
Female	2121	530	25.0	0.69 (0.61 to 0.79)	0.74 (0.64 to 0.86)	

**Age, years**						
16–24	96	16	16.7	0.46 (0.27 to 0.80)	0.48 (0.27 to 0.86)	<0.001
25–34	123	37	30.1	0.99 (0.67 to 1.49)	1.01 (0.66 to 1.55)	
35–44	235	52	22.1	0.66 (0.47 to 0.91)	0.67 (0.48 to 0.95)	
45–54	539	138	25.6	0.80 (0.64 to 1.00)	0.78 (0.62 to 0.99)	
55–64	1153	312	27.1	0.86 (0.72 to 1.02)	0.85 (0.71 to 1.01)	
65–74	1441	435	30.2	Reference	Reference	
75–84	876	279	31.8	1.08 (0.90 to 1.30)	1.07 (0.89 to 1.29)	
≥85	184	80	43.5	1.78 (1.30 to 2.43)	1.64 (1.19 to 2.26)	

**Ethnic group**						
White	4431	1288	29.1	Reference	Reference	0.94
Other	216	61	28.2	0.77 (0.50 to 1.21)	0.99 (0.72 to 1.36)	

**Deprivation group**						
Affluent	987	248	25.1	Reference	Reference	0.001
Deprivation group 2	1023	288	28.2	1.17 (0.96 to 1.42)	1.15 (0.93 to 1.40)	
Deprivation group 3	959	280	29.2	1.23 (1.01 to 1.50)	1.22 (0.99 to 1.50)	
Deprivation group 4	858	247	28.8	1.20 (0.98 to 1.48)	1.19 (0.96 to 1.47)	
Most deprived	820	286	34.9	1.60 (1.30 to 1.96)	1.56 (1.26 to 1.92)	

**TOTAL**	**4647**	**1349**	**29.0**			

a Adjusted for cancer site, age, sex, ethnic group, and deprivation group.

b For adjusted OR. OR = odds ratio.

### Outcome 1: emergency presentation without prior consultations

Among the 4647 emergency presenters, 1349 (29%) reported no prior consultation with a GP (Table 1). Using post-stratification weights (to account for distortions caused by sampling, non-response, and post-sampling mortality), 34% of all emergency presenters with the studied cancers reported no previous consultations.

**Figure 1.**
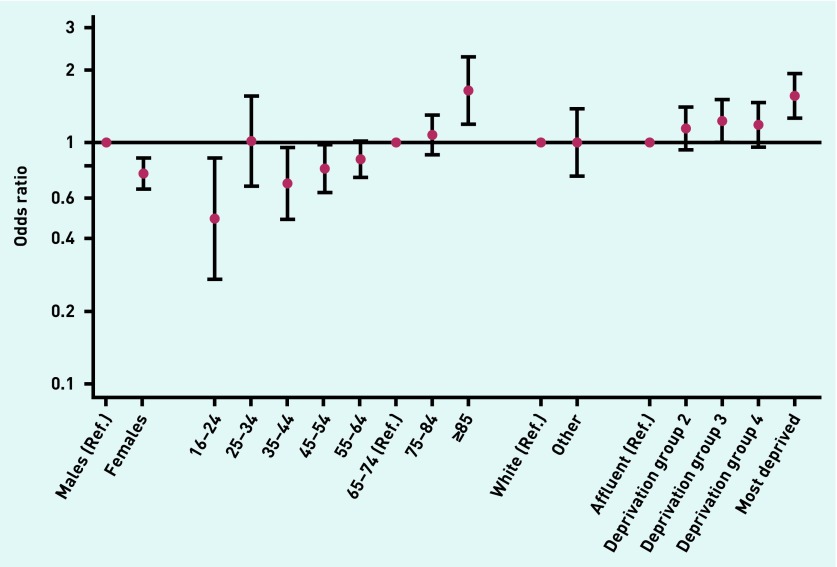
***Adjusted odds ratio for no prior GP consultation among emergency presenters by sex, age group, ethnic group, and deprivation (adjusted for cancer site). Ref. = reference.***

Emergency presenters who were male (32% versus 25% female), older (44% in ≥85-year-olds versus 30% in 65–74-year-olds), and belonged to the more deprived groups (35% versus 25% least deprived) were more likely to report no prior consultations, with little variation by ethnic group. Multivariable logistic regression indicated concordant findings (Table 1, Figure 1).

**Figure 2.**
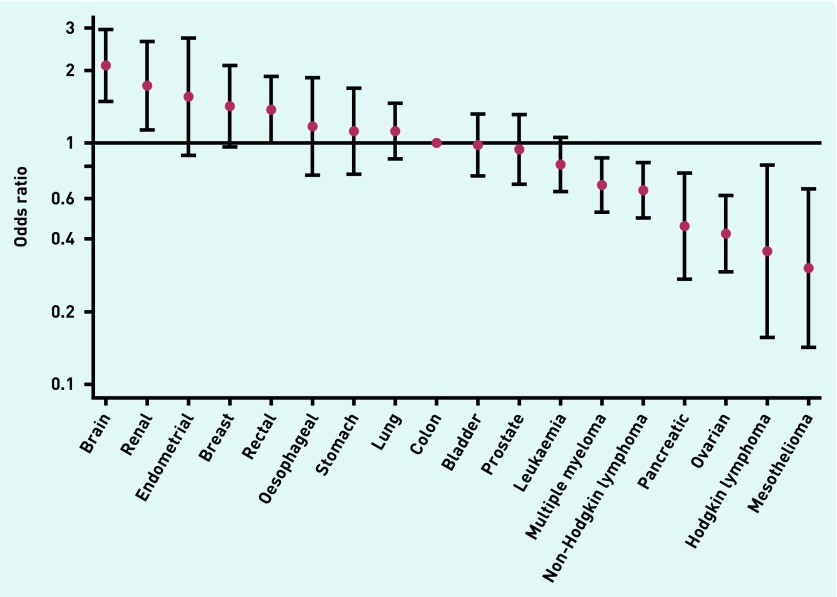
***Adjusted odds ratio for no prior GP consultation among emergency presenters by cancer site (adjusted for sex, age group, ethnic group, and deprivation).***

The proportion of emergency presenters without prior consultation varied greatly by cancer site, as indicated by sevenfold difference in adjusted odds (*P*<0.001). Specifically, emergency presenters with brain, renal, endometrial, or breast cancer were most likely to report no prior consultations (adjusted OR of 2.08, 1.71, 1.54, and 1.42, respectively, using colon cancer as reference); while those with mesothelioma, Hodgkin lymphoma, ovarian, or pancreatic cancer were least likely (adjusted OR of 0.30, 0.35, 0.41, and 0.45, respectively, Table 1, Figure 2).

### Outcome 2: three or more previous consultations (among emergency presenters with at least one consultation)

Among the 3298 emergency presenters with at least one prior consultation, 1356 (41%) had three or more consultations (Table 2). Using post-stratification weights, 35% of all emergency presenters who report that they have consulted previously with a GP would have done so three or more times. This is equivalent to 23% (weighted percentage) of all emergency presenters (independently of prior consultation status).

**Table 2.** Cancer site and sociodemographic predictors of at least three prior GP consultations — among emergency presenters who had consulted at least once (*n* = 3298)

	**At least one prior consultation, *N***	**Three or more prior consultations, *n***	**% (*n*/*N*)**	**Unadjusted OR (95% CI)**	**Adjusted OR (95% CI)^a^**	***P*-value^b^**
**Cancer site**						
Multiple myeloma	394	222	56.3	1.84 (1.41 to 2.40)	1.81 (1.38 to 2.37)	<0.001
Non-Hodgkin lymphoma	402	192	47.8	1.30 (1.00 to 1.70)	1.19 (0.91 to 1.56)	
Lung	255	114	44.7	1.15 (0.85 to 1.56)	1.14 (0.84 to 1.55)	
Prostate	136	50	36.8	0.83 (0.56 to 1.23)	1.05 (0.70 to 1.57)	
Colon	512	211	41.2	Reference	Reference	
Rectal	142	58	40.8	0.98 (0.67 to 1.44)	0.98 (0.67 to 1.44)	
Stomach	73	27	37.0	0.84 (0.50 to 1.39)	0.96 (0.57 to 1.61)	
Ovarian	280	127	45.4	1.18 (0.88 to 1.59)	0.95 (0.70 to 1.29)	
Brain	93	43	46.2	1.23 (0.79 to 1.91)	0.93 (0.59 to 1.47)	
Hodgkin lymphoma	52	26	50.0	1.43 (0.81 to 2.53)	0.87 (0.48 to 1.60)	
Endometrial	35	15	42.9	1.07 (0.54 to 2.14)	0.85 (0.42 to 1.72)	
Renal	57	20	35.1	0.77 (0.44 to 1.37)	0.80 (0.45 to 1.42)	
Pancreatic	98	35	35.7	0.79 (0.51 to 1.24)	0.77 (0.49 to 1.21)	
Oesophageal	53	16	30.2	0.62 (0.33 to 1.14)	0.69 (0.37 to 1.29)	
Bladder	172	44	25.6	0.49 (0.33 to 0.72)	0.61 (0.41 to 0.90)	
Mesothelioma	53	14	26.4	0.51 (0.27 to 0.97)	0.58 (0.31 to 1.11)	
Breast	102	32	31.4	0.65 (0.41 to 1.03)	0.44 (0.27 to 0.71)	
Leukaemia	389	110	28.3	0.56 (0.42 to 0.75)	0.41 (0.30 to 0.55)	

**Sex**						
Male	1707	644	37.7	Reference	Reference	<0.001
Female	1591	712	44.8	1.34 (1.16 to 1.54)	1.35 (1.15 to 1.59)	

**Age, years**						<0.001
16–24	80	38	47.5	1.45 (0.92 to 2.29)	2.22 (1.34 to 3.66)	
25–34	86	43	50	1.60 (1.03 to 2.49)	2.04 (1.27 to 3.29)	
35–44	183	104	56.8	2.11 (1.53 to 2.90)	2.71 (1.92 to 3.81)	
45–54	401	188	46.9	1.41 (1.12 to 1.78)	1.51 (1.18 to 1.93)	
55–64	841	376	44.7	1.29 (1.07 to 1.56)	1.30 (1.07 to 1.58)	
65–74	1006	387	38.5	Reference	Reference	
75–84	597	192	32.2	0.76 (0.61 to 0.94)	0.78 (0.62 to 0.97)	
≥85	104	28	26.9	0.59 (0.38 to 0.93)	0.67 (0.42 to 1.06)	

**Ethnic group**						
White	3143	1275	40.6	Reference	Reference	0.017
Other	155	81	52.3	1.60 (1.16 to 2.22)	1.52 (1.08 to 2.13)	

**Deprivation group**						
Affluent	739	316	42.8	Reference	Reference	0.380
Deprivation group 2	735	300	40.8	0.92 (0.75 to 1.14)	0.94 (0.76 to 1.16)	
Deprivation group 3	679	253	37.3	0.79 (0.64 to 0.98)	0.80 (0.64 to 1.00)	
Deprivation group 4	611	254	41.6	0.95 (0.77 to 1.18)	0.94 (0.75 to 1.18)	
Most deprived	534	233	43.6	1.04 (0.83 to 1.30)	0.95 (0.75 to 1.20)	

**Total**	**3298**	**1356**	**41.1**			

a Adjusted for cancer site, age, sex, ethnic group, and deprivation group.

b For adjusted OR. OR = odds ratio.

**Figure 3.**
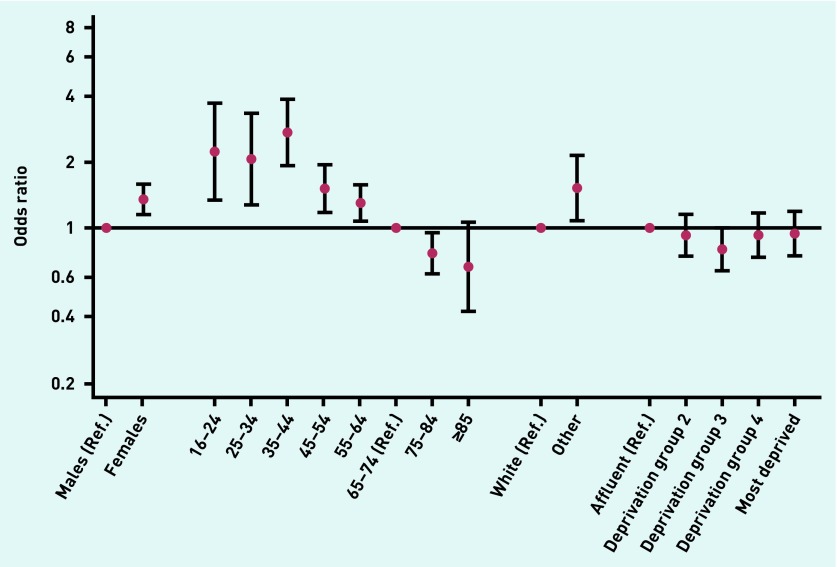
***Adjusted odds ratio for three or more GP consultations among ‘prior consultee’ emergency presenters, by sex, age group, ethnic group, and deprivation (also adjusted for cancer site).*** ***Ref. = reference.***

Three or more consultations were more common in females (45% versus 38% in males), younger patients (48% in 16–24-year-olds versus 39% in 65–74-year-olds), and ethnic minority patients (52% versus 41% for white patients), with little variation by deprivation group. Multivariable logistic regression provided concordant findings (Table 2, Figure 3).

**Figure 4.**
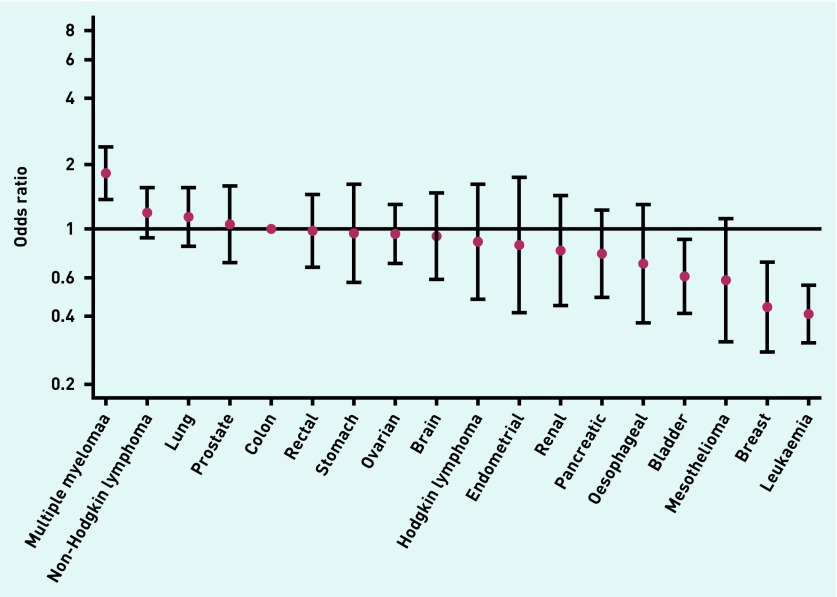
***Adjusted odds ratio for three or more GP consultations among ‘prior consultee’ emergency presenters, by cancer site (adjusted for sex, age group, ethnic group, and deprivation).***

There was large variation by cancer, as indicated by fourfold variation in adjusted odds (*P*<0.001), with three or more consultations being most likely in emergency presenters with multiple myeloma (OR 1.81) and least likely in those with leukaemia (OR 0.41) (Table 2, Figure 4).

#### Sensitivity analysis

Including post-stratification weights in multivariable regression models provided broadly consistent findings (Appendix 4). Although the OR for some cancers changed considerably, these changes were small relative to the width of associated confidence intervals. This highlights that the exact ordering of cancers should not be over-interpreted, rather taken as a broad indication of its position. As expected, weighting increased the standard errors, resulting in some of the smaller variations no longer being significant.

## DISCUSSION

### Summary

About one-third of emergency presenters reported no prior consultation with a GP. Older and more deprived patients, and those with brain cancer, were more likely not to have consulted previously. Of those who had previously seen a GP, younger and ethnic minority patients, and those subsequently diagnosed with ‘harder-to-suspect’ cancers were more likely to have consulted multiple times.

### Strengths and limitations

Patient reports of consultation history were used, which directly relate to perceptions of their own healthcare experience and have high face validity. Patients might overestimate pre-referral consultations compared with information in medical records, but recall inaccuracies would likely introduce random error and could not explain the large observed variations.^17^^,^^18^ Many different cancer sites were examined, enabling stronger inferences to be made on disease factors contributing to emergency presentations.

The sample included emergency presenters with recent hospital treatment, not all emergency presenters among incident cases; however, the weighting of overall proportions took into account compositional differences between the two populations.^14^ Weighting assumed that emergency presenters who died early or did not respond were ‘missing at random’ conditional on their age, sex, deprivation, and cancer site. This assumption may not be true, as emergency presenters in the sample may have different consultation patterns than otherwise similar (in terms of age, sex, deprivation, and cancer site) emergency presenters among incident cases. The potential for such bias is limited, however, as only 6% of all patients died between treatment and survey mail out, and the survey had a relatively high response rate (67%).^14^ Previous related research has shown the effect of sociodemographic factors varying by cancer site, but power considerations meant that such interactions could not be considered in this study.^4^^,^^15^

While the present findings indicate that two-thirds of emergency presenters had at least one prior primary care consultation, previous analysis indicates that GPs are directly involved (that is, through direct emergency referral to hospital services) in one-third of all emergency presentations.^11^ Assuming that emergency presenters who were referred to hospital as emergencies by their GP would consider such involvement to be a prior consultation, the combined interpretation of these figures would indicate that at least one-third of all emergency presenters have had prior contact with GPs but were not referred as emergencies. With the present data, it is impossible to establish the proportion of emergency presentations generated by direct emergency GP referrals.

### Comparison with existing literature

Prior evidence indicates that few emergency presenters had no prior primary care consultations, but is restricted to only three cancers (ovarian, lung, and colorectal) and dominated by medical record studies not examining whether the consultations were on relevant symptoms, and whether they were single or multiple.^2^^,^^19^^–^24

Males, older patients, and the most deprived emergency presenters were more likely to report no prior consultations, possibly reflecting a higher concentration of practical, cognitive, or emotional barriers to presentation in these patient groups.^25^^–^28 It has been further suggested that dementia syndromes may be implicated in emergency presentation in some older patients.^29^ Some deprived patients may prefer using emergency services for regular care, which may also partly explain the findings.^30^

It has long been hypothesised that patients with brain cancer have a relatively high proportion of emergency presentations (>60%) because they often first present with seizures, without prodromal non-acute symptoms. The present findings support this hypothesis, which remains otherwise poorly studied.

It is important to note that the number of pre-referral consultations before a cancer diagnosis cannot be assumed to be a measure of diagnostic quality per se for individual patients. In some patients multiple consultations may represent appropriate care, including, for example, if there is a patient preference for non-referral during earlier consultations, or because of the need to review results of investigations ordered. However, in some patients (particularly when an emergency presentation has ensued), multiple consultations may represent potential for missed diagnostic opportunities. Where such judgements are possible, they can only be made after thorough case note review and the consideration of the presenting symptoms.^31^

### Implications for research and policy

Contrary to suggestions that emergency presentations represent ‘failures of primary care’, the present findings suggest that many emergency presenters have no prior contact with primary care and emphasise the potential influence of psychosocial patient factors.^32^ The fact that males, older patients, and more deprived emergency presenters are more likely not to have consulted provides opportunities for targeting of general public health education interventions about cancer symptoms.^24^

One-quarter of all emergency presenters have three or more prior GP consultations. As hypothesised, among emergency presenters with at least one prior consultation, those with ‘harder-to-suspect’ cancers (such as multiple myeloma and lung cancer) or characteristics associated with greater diagnostic difficulty (for example young age) were more likely to have had multiple consultations.^15^ In these patients, emergency presentations seem to reflect the challenges of suspecting cancer when symptoms are vague, and/or the baseline risk of cancer is low.

Among emergency presenters with breast and endometrial cancer (who had consulted previously), 21% and 33% had three or more consultations, respectively. These proportions are appreciably higher compared with those observed in a typical female with either cancer (7% and 17%, respectively).^15^ This suggests that emergency presenters with ‘easy-to-suspect’ cancers (such as breast and endometrial cancer) may tend to have atypical symptomatic presentations.

Consistent with the study hypotheses, patients with leukaemia had the lowest odds of three or more consultations among prior consultee emergency presenters, possibly reflecting direct emergency referrals by GPs after abnormal full blood count tests.

Generally, interventions aimed at improving diagnostic timeliness after presentation may also reduce emergency presentations.^33^ Such interventions may include greater use of clinical decision-support tools, development of point-of-care tests, and accelerated diagnostic care pathways to specialist assessment and imaging or endoscopic investigations.^34^^,^^35^

In conclusion, against suggestions that emergency presentations represent missed diagnoses, about one-third of emergency presenters have no prior GP consultations, and only about one-third experience multiple consultations. Both disease (for example cancer site and symptom signature) and patient factors (for example, lower levels of symptom awareness in older and more deprived patients) are likely to be implicated in emergency presentations without prior consultation. The findings can guide future research and policies, focusing on public health education campaigns (for example, aimed at changing patient awareness, beliefs and behaviour, particularly in higher risk groups), or healthcare (diagnostic safety) interventions, variably targeting different patient groups and symptomatic presentations.

## Figures and Tables

**Figure:**
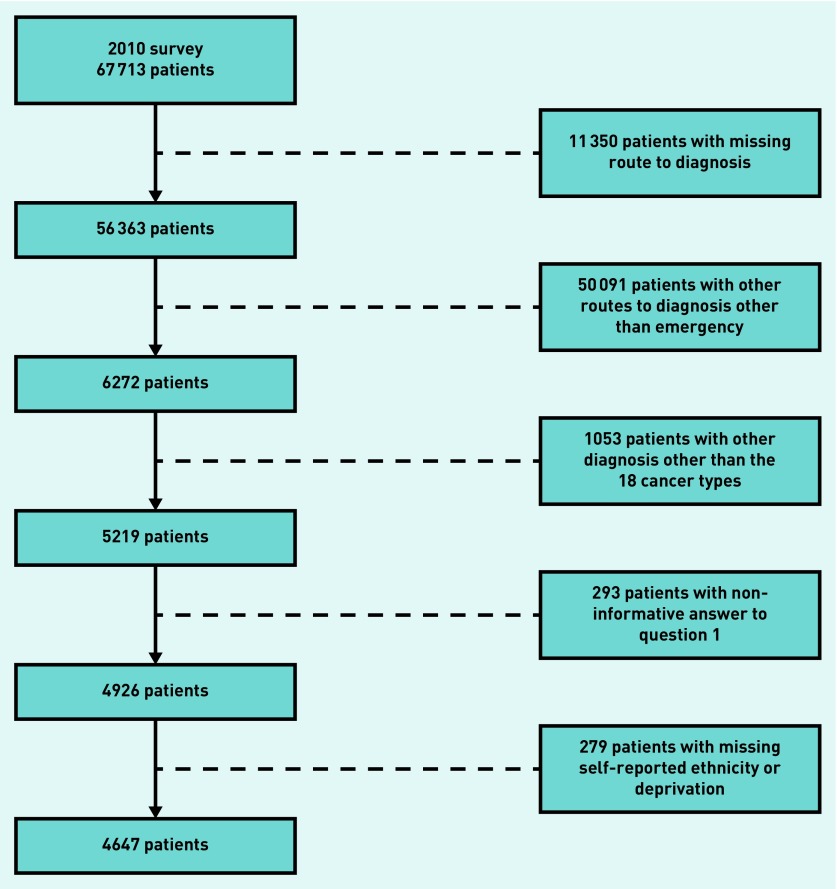
***Sample derivation flow chart.***

## Appendix 1. Linkage method used to assign emergency presentation status

**Table:**

Linkage was carried out previously by the Public Health England National Cancer Intelligence Network to support public reporting of data on cancer patient experience.^a^ Records of English Cancer Patient Experience Survey (CPES) 2010 responders were matched using NHS number to the National Cancer Data Repository (NCDR) 2010 table. Of the 67 713 responders to CPES, 64 815 (95.7%) were matched to 82 788 tumours in the NCDR dataset, with some patients being matched to multiple tumours. A total of 2898 (4.3%) could not be matched to a tumour in the NCDR dataset. Among matched cases, linkage validity was checked using date of birth and found to be perfect in most cases, with plausible partial matches in nearly all of the non-perfectly matched cases. ‘Routes to Diagnosis’ codes were assigned using the most recent tumour in the NCDR dataset with a diagnostic route code. No diagnostic route was available for 8452 of CPES responders matched to the NCDR dataset, leaving 56 363 CPES records (83.2% of all CPES records). A substantial proportion of unlinked cases relate to diagnoses outside of the period covered by the ‘Routes to Diagnosis’ source (2006–2010), with 43% of unlinked responders indicating that their cancer was diagnosed >5 years ago.

a Quality Health. 2014 National Cancer Patient Experience Survey National Report and Related Data. https://www.quality-health.co.uk/resources/surveys/national-cancer-experience-survey/2014-national-cancer-patient-experience-survey/2014-national-cancer-patient-experience-survey-national-reports (accessed 20 Mar 2017).

## Appendix 3. Mean post stratification weights

**Table:**

	**Emergency presenters by variable category stratum in the study sample, % (*N*= 4647)^a^**	**Emergency presenters by variable category stratum among incident emergency presenter cases 2006–2010, % (*N*= 235 324)**	**Mean post stratification weight^b^**
**Cancer**			
Lung	8.4	26.6	3.18
Pancreatic	2.5	7.0	2.75
Stomach	2.5	4.3	1.70
Oesophageal	1.9	3.0	1.64
Renal	2.2	3.4	1.55
Prostate	4.5	6.5	1.45
Brain	3.7	5.2	1.39
Mesothelioma	1.3	1.6	1.21
Breast	3.4	3.8	1.13
Endometrial	1.2	1.1	0.91
Colon	16.2	13.2	0.81
Rectal	5.1	3.5	0.69
Bladder	5.7	3.4	0.60
Ovarian	7.0	3.7	0.53
Non-Hodgkin lymphoma	11.1	5.5	0.49
Leukaemia	11.2	4.9	0.44
Hodgkin lymphoma	1.3	0.5	0.40
Multiple myeloma	11.0	2.9	0.26

**Sex**			
Male	54.4	53.4	0.98
Female	45.6	46.6	1.02

**Age, years**			
16–24	2.1	1.7	0.33
25–34	2.6	0.9	0.45
35–44	5.1	2.2	0.49
45–54	11.6	5.6	0.57
55–64	24.8	14.0	0.75
65–74	31.0	23.3	0.76
75–84	18.9	32.5	1.74
≥85	4.0	19.8	4.91

**Deprivation group**			
Least deprived	21.2	16.1	0.78
Deprivation group 2	22.0	19.4	0.83
Deprivation group 3	20.6	20.8	1.00
Deprivation group 4	18.5	21.6	1.20
Most deprived	17.6	22.0	1.27

The stratification weights are standardised so that values >1 mean the emergency presentation of a type of cancer is under-represented in the English Cancer Patient Experience Survey (CPES) (and vice versa for values <1). These weights can be conceptualised as indicating the number of incident patients with cancer who present as emergencies in the population represented by each emergency presenter in the analysis sample.

a These are the CPES 2010 responders who were diagnosed through emergency presentation.

b *The weights are calculated as follows:*
The number was counted of CPES responders flagged as emergency presenters in the linked Routes to Diagnosis (RTD) 2006–2010 dataset for each cancer by sex, by age group, by deprivation strata (a total of 1440 strata). The number was counted of emergency presenters in the population-based RTD dataset for each cancer by sex, by age group, by deprivation strata (a total of 1440 strata). Where the number was zero in CPES but not in RTD, strata were merged across all deprivation groups (of responders with the same cancer diagnosis, sex, and age group). After merging on deprivation there were still 46 cancer by sex by age strata with zero count in CPES and non-zero count in RTD. These strata were merged such that a stratum containing zero count in CPES was merged with the stratum of the most populous adjacent age group (of responders with the same sex and cancer diagnosis). The weights for each of the 786 final strata were calculated by dividing the number of cases in the RTD dataset by the number of responders. The resulting weights were applied to each responder in the strata. The weights were then normalised such that the mean weight among the analysis sample was = 1.

## Appendix 4. Comparison of adjusted OR from weighted (sensitivity analysis) and unweighted (as used in main analysis) logistic regression

**Table:**

	**No prior GP consultation**			**Three or more prior GP consultations**	
	
	**Unweighted OR: as used in main analysis (95% CI)**	**Weighted OR: sensitivity analysis (95% CI)**		**Unweighted OR: as used in main analysis (95% CI)**	**Weighted OR: sensitivity analysis (95% CI)**
**Cancer diagnosis**			**Cancer diagnosis**		
Brain	2.08 (1.47 to 2.94)	1.17 (0.64 to 2.15)	Multiple myeloma	1.81 (1.38 to 2.37)	1.44 (0.97 to 2.12)
Renal	1.71 (1.12 to 2.62)	0.88 (0.43 to 1.81)	Non-Hodgkin lymphoma	1.19 (0.91 to 1.56)	1.55 (1.04 to 2.32)
Endometrial	1.54 (0.88 to 2.72)	0.79 (0.29 to 2.15)	Lung	1.14 (0.84 to 1.55)	0.96 (0.63 to 1.48)
Breast	1.42 (0.97 to 2.07)	1.16 (0.59 to 2.29)	Prostate	1.05 (0.70 to 1.57)	0.92 (0.52 to 1.60)
Rectal	1.37 (1.01 to 1.86)	1.30 (0.85 to 1.97)	Colon	Reference	Reference

Oesophageal	1.16 (0.73 to 1.85)	0.70 (0.37 to 1.33)	Rectal	0.98 (0.67 to 1.44)	1.03 (0.60 to 1.77)
Stomach	1.11 (0.74 to 1.67)	0.99 (0.54 to 1.81)	Stomach	0.96 (0.57 to 1.61)	1.17 (0.55 to 2.48)
Lung	1.11 (0.85 to 1.44)	1.10 (0.71 to 1.71)	Ovarian	0.95 (0.70 to 1.29)	1.36 (0.75 to 2.46)
Colon	Reference	Reference	Brain	0.93 (0.59 to 1.47)	0.94 (0.43 to 2.04)
Bladder	0.97 (0.72 to 1.31)	0.73 (0.47 to 1.11)	Hodgkin lymphoma	0.87 (0.48 to 1.60)	1.03 (0.43 to 2.49)
Prostate	0.93 (0.67 to 1.30)	0.94 (0.57 to 1.55)	Endometrial	0.85 (0.42 to 1.72)	0.67 (0.23 to 1.96)
Leukaemia	0.81 (0.62 to 1.05)	0.69 (0.40 to 1.17)	Renal	0.80 (0.45 to 1.42)	0.67 (0.31 to 1.48)
Multiple myeloma	0.66 (0.51 to 0.86)	0.80 (0.49 to 1.29)	Pancreatic	0.77 (0.49 to 1.21)	0.88 (0.45 to 1.70)
Non-Hodgkin lymphoma	0.63 (0.48 to 0.81)	0.51 (0.35 to 0.74)	Oesophageal	0.69 (0.37 to 1.29)	0.71 (0.32 to 1.55)
Pancreatic	0.45 (0.27 to 0.74)	0.35 (0.16 to 0.75)	Bladder	0.61 (0.41 to 0.90)	0.62 (0.36 to 1.05)
Ovarian	0.41 (0.29 to 0.60)	0.21 (0.12 to 0.36)	Mesothelioma	0.58 (0.31 to 1.11)	0.47 (0.21 to 1.05)
Hodgkin lymphoma	0.35 (0.15 to 0.80)	0.21 (0.08 to 0.55)	Breast	0.44 (0.27 to 0.71)	0.41 (0.20 to 0.85)
Mesothelioma	0.30 (0.14 to 0.64)	0.43 (0.12 to 1.49)	Leukaemia	0.41 (0.30 to 0.55)	0.45 (0.26 to 0.79)

**Sex**			**Sex**		
Male	Reference	Reference	Male	Reference	Reference
Female	0.74 (0.64 to 0.86)	0.83 (0.61 to 1.13)	Female	1.35 (1.15 to 1.59)	1.33 (0.99 to 1.79)

**Age, years**			**Age, years**		
16–24	0.48 (0.27 to 0.86)	0.49 (0.24 to 1.03)	16–24	2.22 (1.34 to 3.66)	2.47 (1.29 to 4.73)
25–34	1.01 (0.66 to 1.55)	0.96 (0.55 to 1.68)	25–34	2.04 (1.27 to 3.29)	2.24 (1.17 to 4.28)
35–44	0.67 (0.48 to 0.95)	0.64 (0.38 to 1.08)	35–44	2.71 (1.92 to 3.81)	1.25 (0.67 to 2.33)
45–54	0.78 (0.62 to 0.99)	0.79 (0.55 to 1.14)	45–54	1.51 (1.18 to 1.93)	1.40 (0.93 to 2.12)
55–64	0.85 (0.71 to 1.01)	0.84 (0.66 to 1.07)	55–64	1.30 (1.07 to 1.58)	1.39 (1.05 to 1.82)
65–74	Reference	Reference	65–74	Reference	Reference
75–84	1.07 (0.89 to 1.29)	1.02 (0.78 to 1.35)	75–84	0.78 (0.62 to 0.97)	0.73 (0.53 to 1.02)
≥85	1.64 (1.19 to 2.26)	1.49 (0.85 to 2.60)	≥85	0.67 (0.42 to 1.06)	0.54 (0.30 to 0.98)

**Ethnic group**			**Ethnic group**		
White	Reference	Reference	White	Reference	Reference
Other	0.99 (0.72 to 1.36)	0.91 (0.58 to 1.46)	Other	1.52 (1.08 to 2.13)	1.54 (0.90 to 2.64)

**Deprivation group**			**Deprivation group**		
Affluent	Reference	Reference	Affluent	Reference	Reference
Deprivation group 2	1.15 (0.93 to 1.40)	1.19 (0.83 to 1.71)	Deprivation group 2	0.94 (0.76 to 1.16)	1.03 (0.70 to 1.51)
Deprivation group 3	1.22 (0.99 to 1.50)	1.49 (0.99 to 2.25)	Deprivation group 3	0.80 (0.64 to 1.00)	0.70 (0.48 to 1.03)
Deprivation group 4	1.19 (0.96 to 1.47)	1.28 (0.86 to 1.89)	Deprivation group 4	0.94 (0.75 to 1.18)	1.24 (0.82 to 1.88)
Most deprived	1.56 (1.26 to 1.92)	1.43 (0.94 to 2.20)	Most deprived	0.95 (0.75 to 1.20)	0.81 (0.54 to 1.21)

*Spearman correlation coefficients for OR for cancer diagnosis between weighted and unweighted* = *0.79 for ‘no prior consultation’ and* = *0.83 for three or more prior GP consultations. Note: The primary interest is in comparing point estimates obtained from the main and the sensitivity analysis, rather than CI and P-values, as by nature the use of weights increases standard errors.*
